# Aggregative adherence fimbriae form compact structures as seen by SAXS

**DOI:** 10.1038/s41598-023-42079-0

**Published:** 2023-10-02

**Authors:** Rie Jønsson, Alexander Björling, Søren Roi Midtgaard, Grethe Vestergaard Jensen, Nicholas Skar-Gislinge, Lise Arleth, Steve Matthews, Karen Angeliki Krogfelt, Håvard Jenssen

**Affiliations:** 1 Department of Science and Environment, Roskilde University, 4000 Roskilde, Denmark; 2 MAX IV Laboratory, Lund University, Box 117, 221 00 Lund, Sweden; 3 Niels Bohr Institute, Universitetsparken 5, 2100 Copenhagen, Denmark; 4 Department of Life Sciences, Centre for Structural Biology, Imperial College London, South Kensington, London, UK

**Keywords:** Bacteriology, SAXS

## Abstract

Bacterial colonization is mediated by fimbriae, which are thin hair-like appendages dispersed from the bacterial surface. The aggregative adherence fimbriae from enteroaggregative *E. coli* are secreted through the outer membrane and consist of polymerized minor and major pilin subunits. Currently, the understanding of the structural morphology and the role of the minor pilin subunit in the polymerized fimbriae are limited. In this study we use small-angle X-ray scattering to reveal the structural morphology of purified fimbriae in solution. We show that the aggregative fimbriae are compact arrangements of subunit proteins Agg5A + Agg3B which are assembled pairwise on a flexible string rather than extended in relatively straight filaments. Absence of the minor subunit leads to less compact fimbriae, but did not affect the length. The study provides novel insights into the structural morphology and assembly of the aggregative adherence fimbriae. Our study suggests that the minor subunit is not located at the tip of the fimbriae as previously speculated but has a higher importance for the assembled fimbriae by affecting the global structure.

## Introduction

The initial phase of bacterial colonization depends on the ability to attach to a surface. The attachment of the bacteria is mediated by the fimbriae, which are located on the surface of the bacteria. Enteroaggregative *E. coli* (EAEC) is an emerging pathogen, which has been recognized as a major cause of diarrhea worldwide^1–4^. The pathogenesis of EAEC is determined by its ability to colonize the intestinal tract mediated by the aggregative adherence fimbriae (AAFs)^5–9^. Aggregative adherence fimbriae belong to the chaperone-usher (CU) family of adhesins, a highly divergent pathway common to many gram-negative bacteria. The CU pathway is used for assembly and secretion of the fimbriae, and all members harbor a periplasmic chaperone, an outer membrane usher protein, and at least one fimbrial subunit^10–13^. Currently, five different AAFs variants have been genetically characterized, in which all fimbrial clusters encodes 4 genes that confer a “stacked brick” adhesion pattern between the bacterial cells and the eukaryotic cells^6,7,9,14,15^. Previous transmission electron microscopy (TEM) pictures of AAF/I, AAF/II and AAF/III show that the AAFs are long, flexible filaments with estimated diameters of 3–5 nm^6,14^.

The AAFs are composed of two pilin subunits, for AAF/V the major subunit is Agg5A and the minor subunit Agg3B^9^. The two pilin subunits harbor an immunoglobulin‐like fold which lacks the final antiparallel G‐strand, resulting in an unstructured N‐terminal hydrophobic groove^16,17^. When secreted into the periplasm, the pilin domains form a complex with the chaperone (donor strand complementation), thereby stabilizing the subunits and preventing them from auto‐assembly. At the usher, each pilin subunit is inserted into a neighboring pilin subunit, replacing the G-strand from the chaperone with G-strands from neighboring pilin subunit (donor strand exchange), resulting in a polymerized fimbriae which is secreted through the outer membrane^10–12,17^. A study done by Andrea Berry et al., suggests the minor subunit forms the tip of the fimbriae, by accepting the donor strand (DS) from the terminal major pilin subunit^17^. Deletion of the minor pilin subunit has shown not to have an effect on adhesion but instead, the minor pilin subunit has been shown to be the contributor to the release of proinflammatory cytokines from mammalian cells^18–20^.

The monomeric structure of Agg5A and other AAF variants have been resolved by NMR, but these are monomers rather than long polymerized fimbriae^16,17^. In order to understand the interactions between the EAEC AAF fimbriae and the host receptors, it is crucial to unravel the structural architecture of the fimbriae and the role of the minor pilin subunit in the fimbrial polymerization.

In this study we use small-angle X-ray scattering (SAXS) to characterize the polymerized fimbriae in solution from a strain harboring the full fimbriae cluster expressed from a constitutive promoter as well as a fimbriae mutant (Δ*Agg3B*) where the Agg3B subunit has been deleted. Δ*Agg3B* was still able to adhere to plastic surface and to form biofilms, suggesting that Δ*Agg3B* fimbriae are functional. The SAXS data suggest that the AAF/V are flexible sticky chains which fold together in solution and that the presence of Agg3B affects the global conformations of the fimbriae. Studies of long polymerized proteins in solution are limited and SAXS based findings contributes to new knowledge on the structural assembly of the AAFs.

## Results

**Figure 1:**
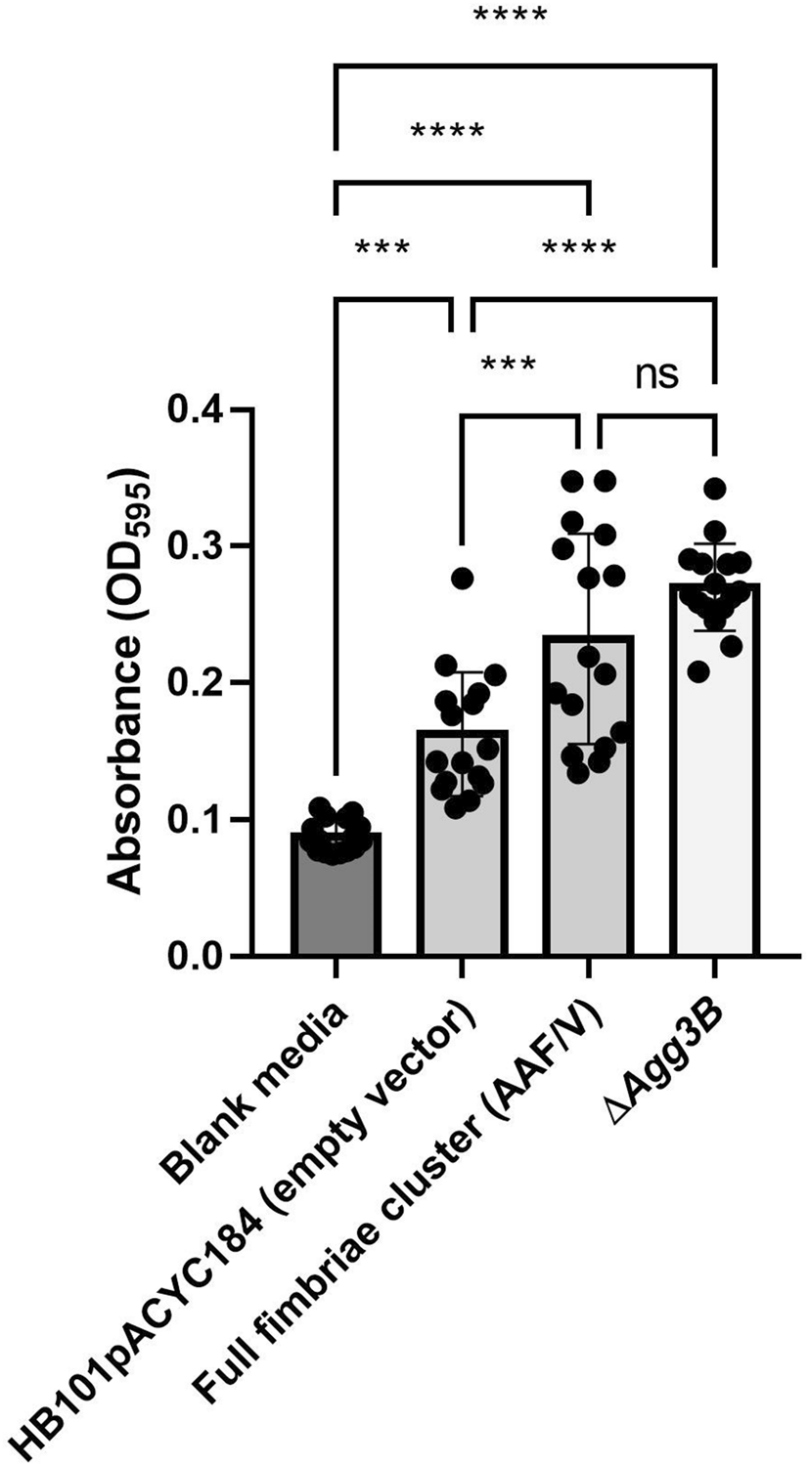
Deletion of minor pilin subunit (Δ*Agg3B*) does not affect adhesion and biofilm formation. The full fimbriae cluster and Δ*Agg3B* was inserted into *E. coli* HB101 and adhesion to plastic surface was performed. The results are represented as the means ± standard errors of the means for eight replicates and represent one of three independent experiments performed with similar results. ****P < 0.0001; ***P < 0.001.

The full fimbrial cluster (AAF/V) was cloned as previously described into the plasmid vector pACYC184 and expressed in the non-fimbriated *E. coli* strain HB101^9^. To test the involvement of the minor pilin subunit in the pilus polymerization, the minor pilin subunit was deleted from the fimbrial cluster (Δ*Agg3B*) and tested for its functionality by biofilm formation in the microtiter plate assay^21^. The bacteria were incubated in microtiter plates for 24 h at 37 °C and then the biofilm was visualized by crystal-violet staining. The results showed that there was no statistical difference between the full fimbriae cluster and Δ*Agg3B* (*P* = 0.12), suggesting that the minor pilin subunit of AAF/V is not attributable for the adhesive phenotype of AAF/V and that Δ*Agg3B* expresses functional fimbriae (Fig. 1).

### Purification of whole fimbriae, dsc-Agg5A and dsc-Agg5AB

**Figure 2:**
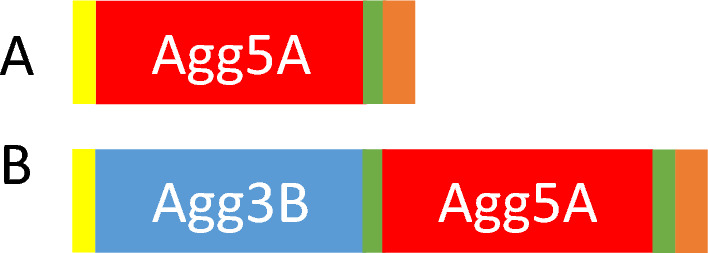
Schematic representation of the two donor complemented constructs. (**A**) dsc-agg5A, (**B**) chimeric dsc-Agg5AB construct. N-terminal his tag is colored in yellow, DNKQ linker in green and the N-terminal extension in orange.

Whole polymerized fimbriae from the wildtype and Δ*Agg3B* were purified as large soluble aggregates that eluted in a broad peak during size exclusion chromatography. Since monomeric subunits lack the antiparallel G-strand which makes them uncapable of folding without a neighboring subunit, donor strand complementation (dsc) of the fimbrial subunits was necessary^17^. To elucidate the role of the minor pilin subunit (Agg3B), a chimeric construct containing both the major pilin subunit (Agg5A) and minor pilin subunit (Agg3B) was created. Mature Agg5B was fused to the N-terminal of the mature Agg5A with an intervening 10-residue linker (DNKQNATAVA), resulting in the fused construct dsc-Agg5AB.The N-terminal donor strand of Agg5A was fused to the C-terminus of Agg5A with a 4-residue linker (DNKQ) (Fig. 2).

Both proteins were expressed in the *E. coli* cytoplasm and refolded as previously described^16,17^. During purification of the subunits, two bands eluted during gel filtration, corresponding to a monomer and dimer. The monomer fractions were used for the SAXS.

### Structural characterization of fimbriae

**Figure 3:**
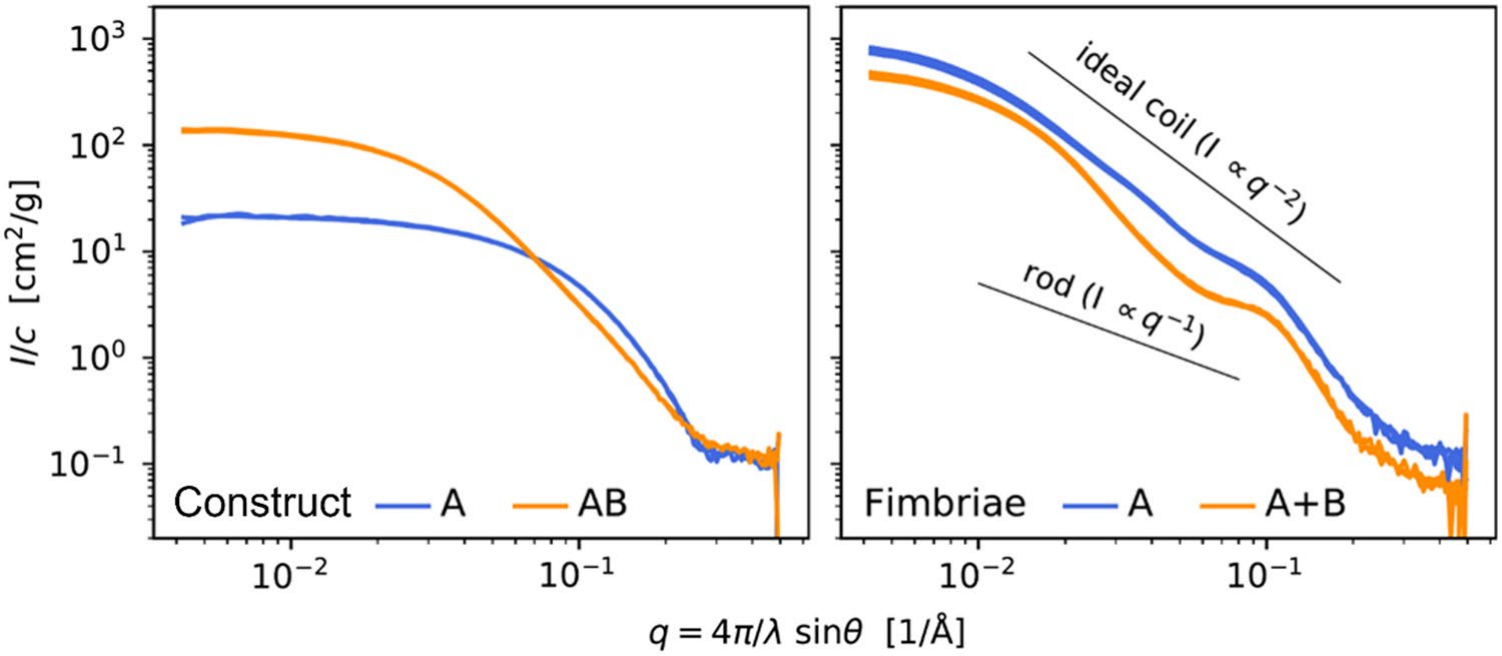
Small angle X-ray scattering data collected and normalized by mass concentration. Curves of different concentrations (shades of blue or orange) are superimposed.

Two fimbriae types were subjected to SAXS analysis, together with engineered subunit samples (Table 1)^17^. Scattering curves obtained from both the constructs and fimbriae samples are plotted and the individual dilution series are superimposed (Fig. 3).

**Table 1:** Sample overview for structural analysis.

Sample	Theoretical *M* (kDa)	Experimental *M* (kDa)	*N*	*Rg* (Å)	Dilutions	Concentration (mg/mL)
Dsc-Agg5A	16.5 kDa	33	2.0	19–28	1:1, 1:2	5.01
Dsc-Agg5AB	31.2 kDa	213	6.8	46	1:1, 1:2	7.31
Fimbriae Agg5A with Δ*Agg3B deleted*				156	1:1, 1:2, 1:4	7.14
Fimbriae Agg5A + Agg3B				138	1:1, 1:2, 1:4	12.2

The radius of gyration (*R*_*g*_) of a particle is a well-defined measure of the overall size of a single homogeneous particle of any shape or form. Guinier analysis (Supplementary data) gave estimates of the gyration radii for all four sample types (Table 1).

At low *q*, the curve is leveled off, for the subunit samples, allowing us to determine an estimation of the molecular weight and aggregation number of these two samples (Supplementary material). Though, molecular weight determination using Guinier approximation of SAXS data should allow for a 10% inaccuracy, it clearly illustrates how dsc-Agg5A dimerizes as the dominant species in solution, while the dsc-Agg5AB aggregates into a larger structure with an average size close to 7 subunits. As the dilution curves overlay superimpose perfectly, the assembly is not concentration dependent, indicating a specific or irreversible interaction between dsc-agg5AB. The dimerization of dsc-Agg5A is further corroborated by fitting of the SAXS curves to other filament model molecules (Supplementary Fig. S2, Supplementary Table S1), as well as the NMR structure of dsc-Agg5A (Supplementary Fig. S1), leading up to an ab initio simulation (Supplementary Fig. S3A). When modeling the dimeric structure of dsc-Agg5B, we relied on ab initio techniques guided by the monomer NMR structure of dsc-Agg5A. Though the resolved structures for dsc-Agg5AB do change from prediction to prediction, they are consistently large and elongated (Supplementary Fig. S3B).

The purified fimbriae species aggregate in a slightly different manner, contrary to the subunit’s aggregation pattern. Radii of gyration are larger for the fimbriae Agg5A compared to the fimbriae Agg5A + Agg3B. The latter is shifted down relative the Agg5A fimbriae at the log–log plot (Supplementary Fig. S4). This drop in scattering intensity would normally indicate that the average molar mass of fimbriae Agg5A + Agg3B is lower than the Agg5A fimbriae. This can in principle be a result of structural differences, which then would be expected to disappear at high *q*, resulting in overlapping curve patterns. However, the fact that the curves align parallel at high *q*, indicate an error in the measured concentration, and indeed the nominal concentration in Table 1 is unusually high.

By using SAXS to analyze it, it is possible to characterize the arrangement of the subunits in the filament. However, this does rely on validated models which can be made using idealized structures such as rods, spheres, disks, or random coils, and the power of Monte Carlo simulations. SAXS data can be directly compared to idealized arrangements such as rods, discs, spheres, or random coils, which give rise to characteristic slopes in plots of log(*I*) vs log(*q*). Fimbriae might be expected to form extended rod-like structures in solution, which would give a slope of − 1 in such a plot, however Supplementary Fig. S4 clearly shows that this is not the case. Instead, the overall slope is close to − 2, which would correspond to an ideal random coil^22^. We conclude that purified fimbriae in solution do not extend like rods, but form more compact arrangements.

**Figure 4:**
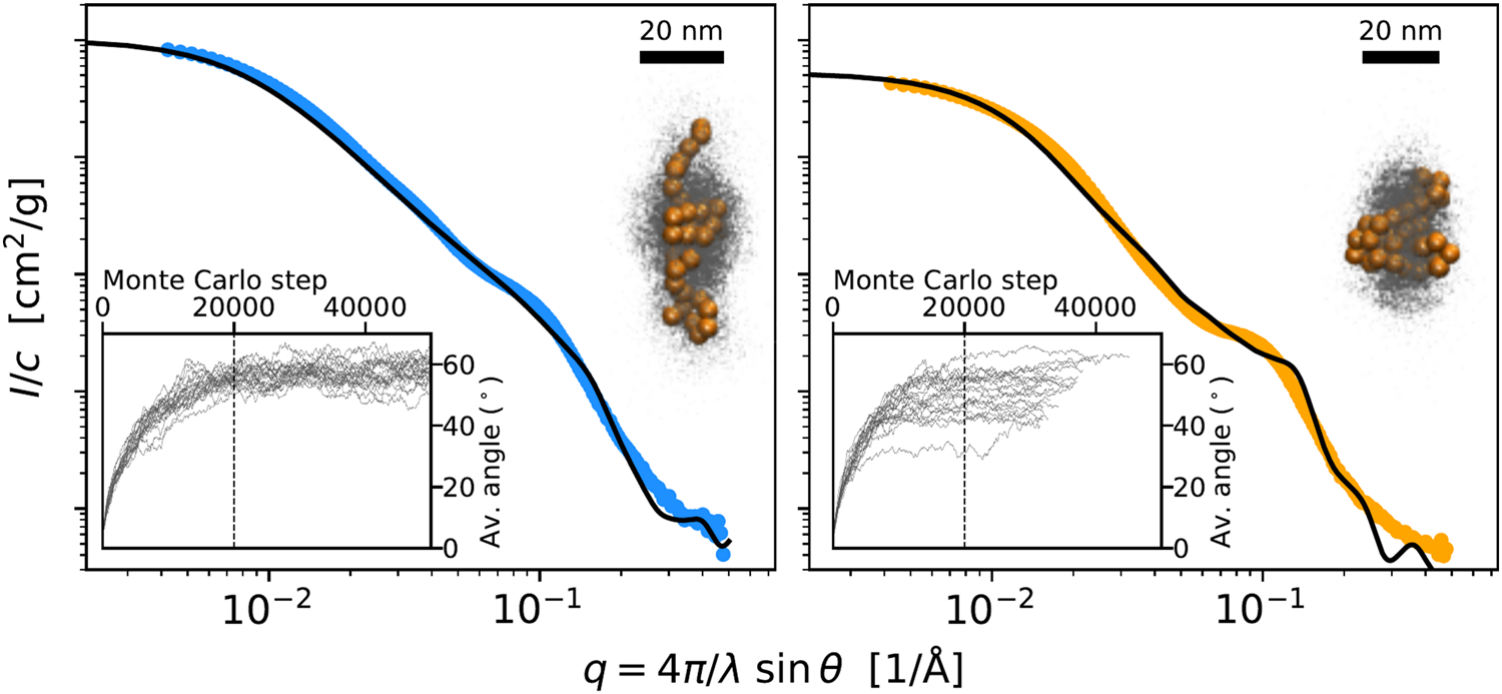
Model fits to Agg5A and Agg5A + Agg3B fimbriae data. The insets show the aligned trajectories (blue dots) and arbitrarily chosen structures (orange chains). Molecular representations are made using VMD version 1.9.3.^23^.

Supplementary Figure S4 shows that the ideal coil model is not a perfect fit, as the curves show features other than the overall slope. Also, that model is not physically relevant, as different parts of the coil would be free to overlap. Therefore, an explicit model of chains of proteins was constructed, where linear chains of subunits are treated as hard spheres which interact with an attractive potential (Supplementary Fig. S5, Supplementary Table S2). The model can be sampled by Monte Carlo simulation (Fig. 4) and can be compared with experimental SAXS data as described in the Supporting Information.

The fitting parameters (Table 2), which also show the dimeric subunit structure used in the modelling, results in a rather flexible chain with *Ө*_*Max*_ = *π*/2, and thus higher flexibilities were not considered. Table 2 also lists *c*/*c*_nom_, the ratio of the fitted concentration to the nominal value listed in Table 1.

**Table 2:** Refined model parameters and the subunit structure used.

	Agg5A	Agg5A + Agg3B	*F*^2^(*q*) model
*N*	40	40	
*d*	50 Å	55 Å	
*Ө*_*Max*_	*π*/2	*π*/2	
*β*	1.1	2	
*c*/*c*_nom_	1.15	0.60	

Molecular representations are made using VMD version 1.9.3.^23^.

Both the Agg5A and Agg5A + Agg3B fimbriae samples give very similar model parameters and arrange in linear assemblies of approximately 40 dimeric subunits, spaced 50 and 55 Å apart, respectively. The major difference between these fimbriae is the degree of stickiness, where the Agg5A + Agg3B fimbriae have a higher tendency to fold together onto themselves, expressed as a (*β*) higher sticking energy in the model (Supplementary Fig. S6). This is what gives both the clearer plateau at intermediate *q* and the smaller radius of gyration. The higher stickiness of fimbriae containing subunit Agg3B is consistent with the aggregation of the Agg5A/Agg3B complex discussed above. Similarly, to stickiness we can model stiffness (Θmax) on chain structure and predicted scattering. As the chain is made stiffer, its scattering pattern transitions from that of the flexible and self-avoiding coil to that of an ideal rod, with a slope of − 1 in the log–log plot, as expected (Supplementary Fig. S7).

## Discussion

The AAFs are a divergent group which has been implicated in multiple phenotypes related to pathogenesis. AAFs have been linked to adherence to a variety of cell lines, ability to form biofilm on biotic and abiotic surfaces, adherence to erythrocytes and stimulation of the proinflammatory cytokine (IL-8)^5,9,18,19,24–26^. Currently, no specific receptor has been identified for AAF/V but a recent study suggests that AAF/II binds to heparan sulfate proteoglycans on human colonoids^5^.

In this study, we investigated the structural characteristics of AAF/V by SAXS. Adherence fimbriae are known to form bundles, and the fact that they fold together into sticky balls in solution is perhaps to be expected^6,7^. Fimbriae naturally sit packed and aligned together on the bacterial surface. It is likely that a propensity for these chains to associate is a necessity to be able to form the bundles, which are often seen in microscopic images. However, the results demonstrated that the different fimbriae types had the ability to assemble into well-defined flexible strings. Furthermore, as concentration variations did not affect the arrangement, unspecific aggregation resulting in miss folding could be excluded. Thus, the results are in support of a biological relevant arrangement of the subunits into longer flexible chains. The results corroborated previous TEM of the AAFs which has demonstrated presence of long flexible fibers dispersed from the bacterial surface. AAF/I and AAF/II have shown to be long and bundled (2–5 nm), whereas AAF/III was most commonly observed in individual filaments^6,7^.

Interpretation of the SAXS data requires use of models, which were generated using simple physical assumptions. The models rest on the key assumption that the known structures of the monomer subunits are intact and do not undergo unfolding as part of the dimerization step. This assumption is supported by structural characterization of other fimbriae types^17^.

We also sought to elucidate the role of the minor pilin subunit by constructing a Δ*Agg3B* mutant and investigate how this affected the polymerized fimbriae. Previous studies have shown that the minor pilin subunit from AAF/II is not attributable for the adhesive phenotype of AAF/II but instead related to induction of the proinflammatory cytokine IL-8^18,19^. No difference was observed between the wildtype and Δ*Agg3B* in biofilm formation, confirming that Δ*Agg3B* mutant is not involved in the adhesion and confirming that the Δ*Agg3B* fimbriae are functional. The fact that the presence of the Agg3B subunit affects the global structure of the fimbriae suggests that it is present to a significant degree in the chains. This observation does not match the hypothesis that Agg3B is only present in one copy at the very tip of the fimbriae to terminate DSC^17^. Such a small presence could not alter the propensity of the entire chain to stick together.

## Materials and methods

### Dsc-Agg5A purification

The dsc-Agg5A was purified as previously described^16^. Briefly, the cells were grown in LB until OD_600_ reached 0.6, following induction by 1 mM IPTG, which was followed by overnight incubation at 37 °C before the cells were harvested by centrifugation. The cells were lyzed by sonication under denaturing conditions before being purified with Ni–NTA (Qiagen). The eluate was first dialyzed against 50 mM sodium acetate pH 5, 50 mM NaCl, 1 M urea, which was followed by a second dialysis against the same buffer but without urea. Agg5a was further purified by gel filtration using a Superdex 75 gel-filtration column (GE Healthcare).

### Dsc-chimeric Agg5AB

A donor strand complemented construct of dsc-Agg5AB was ordered from Genscript. Agg3B was fused to the N-terminal of the mature Agg5A with an intervening 10-residue linker (DNKQNATAVA) (Fig. 2).

The N-terminal donor strand of Agg5A was fused to the C-terminus of Agg5A with a 4-residue linker (DNKQ). Dsc-Agg5AB was cloned into a pQE‐30 plasmid (Qiagen) containing a vector encoded N‐terminal His6 tag and purified according to the same method as described for dsc-Agg5A.

### Construction of Agg5ACD clone

Whole purified fimbriae were obtained from HB101(pDKAAF5) expressing the whole AAF/V cluster as previously described^9^. To obtain a construct without the minor pilin subunit, an Agg5ACD mutant was generated for this study. Briefly, a reverse PCR was carried out on pDKAAF5 with the primers GCGCGCCTCGAGGTATAGTTTTGGGAAGATAACAGTAT and GCGCGCCTCGAGAGATTCCTTCTGCTATATGCATA to delete the B subunit. Amplified DNA was digested with the restriction enzyme *xhoI* and ligated. The correct deletion was verified by sanger sequencing with the primers GCTATAGATAACCCACTGTACAAG and GGTGTATCTGAGTG GATTGTCAGA.

### Microtiter dish biofilm formation assay

Biofilm formation has been shown to correlate with AAF adhesion^9,21^. To test if Δ*Agg3B* was able to express functional fimbriae, the microtiter dish biofilm formation assay was performed as described previously^9,21^.

### Purification of polymerized fimbriae

Purification of whole polymerized fimbriae were purified by the method by Izquierdo et al.^26^. The presence of AAF/V fimbriae was confirmed by SDS-page.

### Small angle X-ray scattering

The SAXS experiments were carried out on the bioSAXS BM29 beamline at the European Synchrotron Radiation Facility (ESRF) in Grenoble, France, using a X-ray wave length of λ = 0.9919 Å and a sample-detector distance of d = 287.2 mm, providing a *q*-range of 0.00423–0.49 Å^−1^.

Scattered X-ray intensity is a function of the angle between the incoming beam and the scattering direction (2*θ*). This angle is most conveniently expressed in terms of the magnitude of the wave momentum transfer (*q*), which in terms of the wavelength (λ) and the half-angle (*θ*) is *q* = (4*π*/*λ*) × sin(*θ*). The quantity *q* carries units of inverse length, and is therefore directly comparable to the length scales present in the sample. All scattering data in this report are presented as angle-dependent intensities, *I*(*q*).

Measurements were performed at 10 °C and data were collected in 20 individual one-second exposures. The individual data frames were merged, scaled to absolute values using water as a reference and background subtracted using the ATSAS software^27^. Four samples were analyzed at different concentrations. Arbitrary constants of 6.2 × 10^–2^ and 2.5 × 10^–2^ cm^2^/g have been respectively subtracted from the A and A + B data to account for inaccuracies in the buffer subtraction. Dsc-Agg5A and dscAgg5AB, were analyzed individually, as well complete fimbriae assembled in the producer strains, either as a fimbria composed of purely subunit Agg5AA or a fimbriae species composed of a mixture of subunit Agg5A and Agg3B (Table 1).

### Supplementary Information

Supplementary Information.

## Data Availability

All data generated or analyzed during this study are included in this published article and its supplementary information files and the following https://doi.org/10.5281/zenodo.7404371.
