# Supplementary material for: Aggregative adherence fimbriae form compact structures as seen by SAXS
Source: Sci Rep. 2023 Oct 2;13:16516. doi: 10.1038/s41598-023-42079-0 (PMC10545799; doi:10.1038/s41598-023-42079-0)
Supplement: Supplementary file 1 — Supplementary Information. [file 41598_2023_42079_MOESM1_ESM.pdf]

SUPPLEMENTARY MATERIAL

Aggregative adherence fimbriae form compact structures as seen by SAXS

Rie Jønsson[1,*], Alexander Björling[2], Søren Roi Midtgaard[3], Grethe Vestergaard Jensen[3], Nicholas Skar-Gislinge[3], Lise Arleth[3], Steve Matthews[4], Karen A. Krogfelt[1], Håvard Jenssen[1,*]

[1]Department of Science and Environment, Roskilde University, 4000 Roskilde, Denmark

[2]MAX IV Laboratory, Lund University, Box 117, 221 00 Lund, Sweden

[3]Niels Bohr Institute, Universitetsparken 5, 2100 Copenhagen, Denmark

[4]Centre for Structural Biology, Department of Life Sciences, Imperial College London, South Kensington, London, United Kingdom

*Corresponding authors: jenssen@ruc.dk and riej@ruc.dk

Materials and methods:

SAXS data collection:

SAXS experiments were performed at the automated BM29 bioSAXS beamline at the European Synchrotron Radiation Facility (ESRF) in Grenoble, France.

Model free analysis:

The radius of gyration (*Rg*) of a particle is a well-defined measure of the overall size of a single homogeneous particle of any shape. In the region $q \leq 1/Rg$, where distances comparable to *Rg* are probed, the intensity follows the simple Guinier approximation (Eq.1),

$$I(q) \propto exp\left(-q^2 R_g^2/3\right) \quad (1)$$

This is seen as a characteristic plateau in the curve at low *q* in a log-log plot. From this relation, radii of gyration can be calculated independently of any structural models or assumptions. For higher angles, the Guinier approximation does not hold. The shape of the scattering pattern *I*(*q*) is then given by the structure of the scattering molecules, as encoded in the so-called form factor (*P*(*q*)). In terms of mass concentration (*c*m) and weight (*M*), the scattering is given by Eq. 2.

$$I(q) = c_m M \frac{(\Delta p_m)^2}{N_A} P(q) \quad (2)$$

where Δp_m is the average excess per-mass scattering length density of proteins compared to water (2.0×10^{10} cm/g), and *N*A is Avogadro's number. However, because $P(q \rightarrow 0) = 1$, the molecular weight of the scattering molecules can be determined in a model-free way from the value of $I_0 = I(q \rightarrow 0)$.

$$M = \frac{I_0}{c_m} \frac{N_A}{(\Delta p_m)^2} \quad (3)$$

Structural characterization of subunits:

In many fibers, subunits are linked together by donor strand complementation where an N-terminal donor strand segment from one subunit binds into a hydrophobic binding pocket in the neighboring subunit. Thus, it is theoretically possible to make spherical permutated constructs of them by engineering part of their C-terminal fragment onto the N-terminal side of

the molecule[1,2]. Though, this in theory should result in monomer subunits, it is apparent from the SAXS study on dsc-Agg5A, despite donor strand complementation (Fig. S1), that it mainly exists as a dimer in solution (original paper Fig. 1, Table 1). These findings align with previous crystal structure studies illustrating formation of self-complementary dimers of homologous proteins[3]. Then, two subunits are stacked on top of each other in a cylindrical fashion. On the other hand, many other homologous structures contain dimeric asymmetric units which are not self-complimentary. Some of these arrangements are listed in Table S1, complemented with their calculated radii of gyration.

Because of the uncertainty in the experimental gyration radius of dsc-Agg5A, none of the models in Table S1 can be unambiguously chosen. The experimental range of *Rg* values essentially allows for all the listed dimeric structures. The same is seen when scattering patterns from the models of Table S1 are calculated and compared to experimental data, as in Fig. S2. There, all the models with exception of the monomeric NMR structure lie close to the experimental curve, though clearly no single model describes the data quantitatively. On the other hand, a weighted average of these model curves readily reproduces experimental data, which suggests that the sample is a mixture of both monomeric and dimeric forms of dsc-Agg5A.

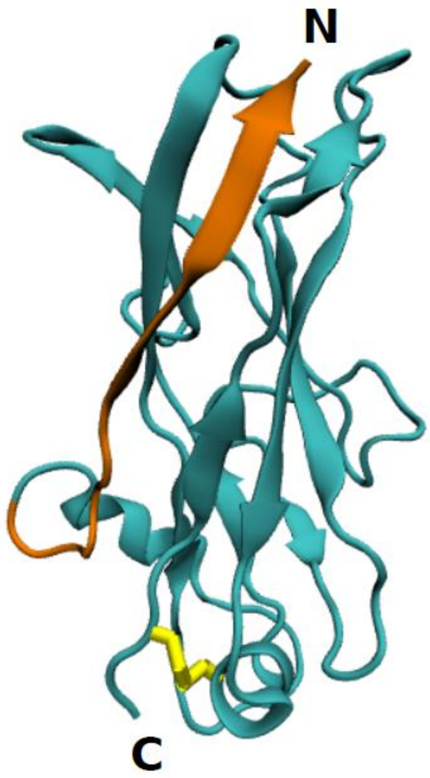

Figure S1 - NMR structure of the modified dsc-Agg5A.

The donor strand, which has been moved from the C-terminal to the N-terminal part of the molecule is marked in orange, while the conserved disulfide bridge is highlighted in yellow[4]. The molecular representation has been generated using Visual Molecular Dynamics VMD 1.9.3.[9]

Table S1: Comparison of the experimental gyration radius to various structures of AAF major pilin subunits. The models rely on four previously reported PDB structures; 5LVY[4], 4OR1, 4PH8[3] and 2AXW[5], and are generated using Visual Molecular Dynamics (VMD) version 1.9.3.[9]

**PDB code**	**Structure**	***Rg***
5LVY	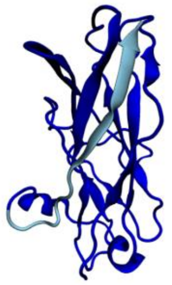	15.7 Å
4OR1	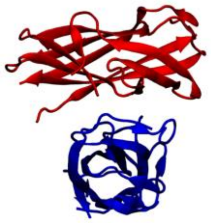	19.3 Å
4PH8	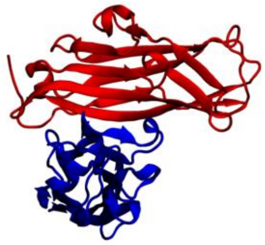	20.7 Å
2AXW	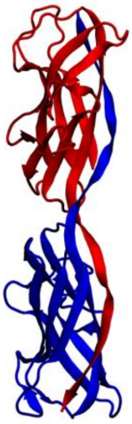	27.0 Å

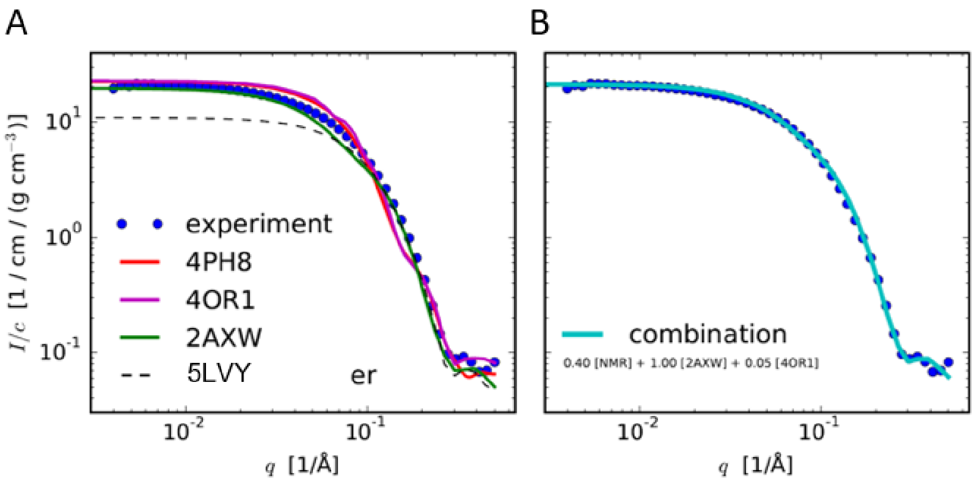

Figure S2 - Scattering data fitted to the structural models.

A Representable PDB structures (Table S1) are used as background for fitting the SAXS.

B By merging the model curves in a linear combination, the model fits perfectly over the experimental curve, after a constant of 4×10^{-2} cm^2/g has been subtracted, to account for the inaccuracies in the buffer subtraction.

An *ab initio* protein simulation is a computational modeling of protein structure and dynamic using a first principle method, involving protein structure prediction, energy calculations, molecular dynamics simulation, analysis and validation[8]. The shape reconstruction of the subunit A data is shown together with the self-complimentary dimer (2AXW) and the monomeric NMR structure (5LVY) (Fig. S3A). The reconstruction shows a dense part equivalent to a monomeric structure, as well as a less dense part, which corresponds to the second half of a dimer. Such a shape is expected for a solution mixture of monomers and dimers. Taken together, the linear combination in Fig. S2 and the *ab initio* shape reconstruction in Fig. S3A, point towards a mixture of a self-complimentary dimer (such as 2AXW) and of the NMR subunit structure. The coefficients in the linear combination gives a rough estimate of the proportions of monomers and dimers. These proportions are normally dependent on concentration, but no such dependence was found in the raw data (see original paper Fig. 3). Possibly, the time scale of equilibration is longer than the few minutes needed to dilute and measure the subunit sample.

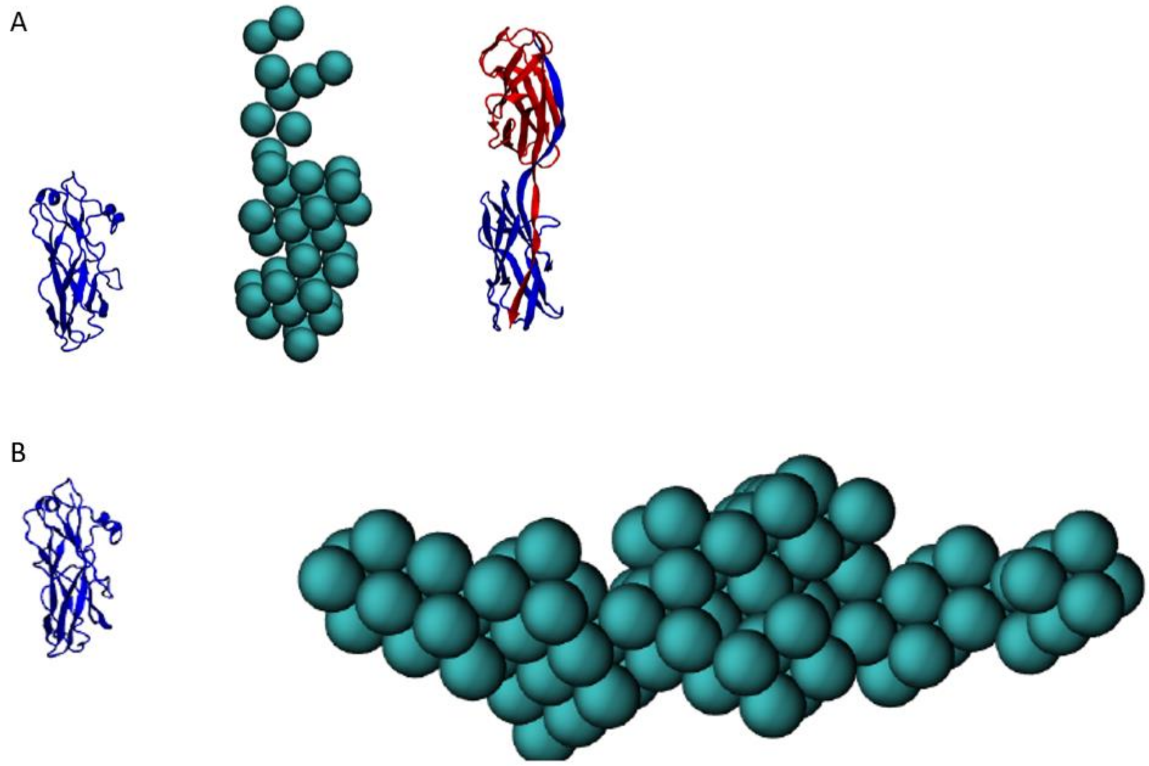

Figure S3 - *Ab initio* structural reconstruction.

A Dsc-Agg5A using balls and space filling, with the complementing NMR structure of the monomer unit in blue ribbon, and the dimeric homologues structure (PDB.2AXW) in red and blue ribbon.

B Dsc-Agg5AB using balls and space filling. All structures are drawn to scale. All structures are drawn to scale. Molecular representations are generated using VMD version 1.9.3.[9]

As discussed above, the dsc-Agg5AB oligomerizes in solution, as seen both in the apparent molecular weight and in the radius of gyration. We have no starting points for describing these assemblies structurally. Thus, an *ab initio* model is presented and the shape reconstruction is in line with the initial results, demonstrating that dsc-Agg5AB oligomerizes (Fig. S3B). The shape is not unique, and different shapes are identified though they consistently are grouped as large and elongated.

Choice of model

SAXS data can be directly analyzed and compared to idealized structures such as rods, spheres, disks, or random coils, to mention a few. These structures give characteristic slopes in plots of log *I* vs. log *q*, which makes validation easy. Fimbriae are expected to adopt rod-like structures, which would correspond to a slope of -1 in such a plot (Fig. S4). However, the fimbriae scattering data demonstrates a slope close to -2, which aligns more with an expected ideal random coil. This is clear evidence that the fimbriae do not form rod-like structures but instead fold into conformations that are more compact.

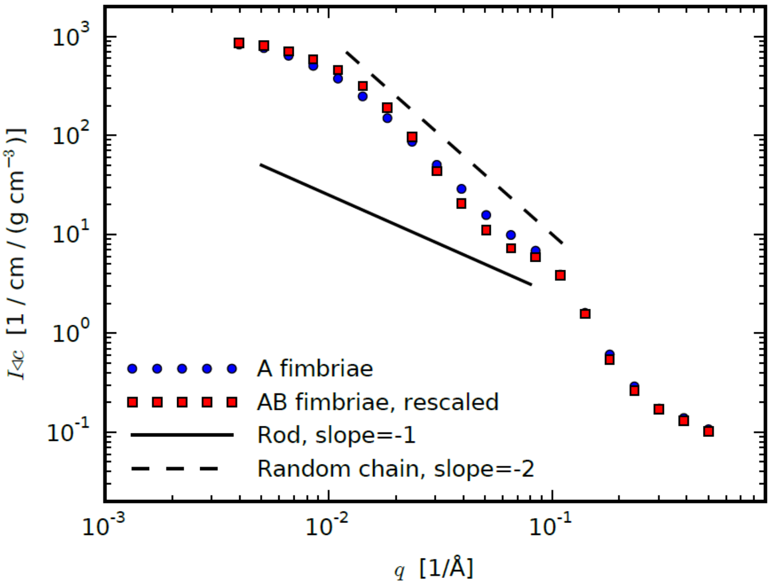

Figure S4 - Fimbriae data compared to the ideal cases of linear rods and random coils. The AB data set has been multiplied by a factor of 2 for easier comparison.

Monte Carlo simulation - Model construction

Interpreting the fimbriae as a chain of subunits, these subunits can be monomers or small assemblies, arranged like beads on a string. The chain's parameters can be varied, and the resulting ensemble of the fimbriae structures can be sampled in a simple Monte Carlo simulation, where each set of chain parameters and a number of structures are obtained from the simulation. Subsequently the X-ray scattering pattern of each structure are calculated and the corresponding chain parameters explaining the structure are derived.

Calculating X-ray scattering for chains of proteins

Given a subunit structure and a chain structure (a list of bead coordinates), the total scattering from the fimbria can be written as a general Debye sum (Eq. 4.)[6],

$$I(q) = \sum_i \times \sum_j \times f_i(q) f_j(q) \frac{\sin q r_{ij}}{q r_{ij}} \qquad (4)$$

where *i* and *j* run over all atoms, f_i is the scattering factor of atom *i*, and r_{ij} is the distance between atoms *i* and *j*. If instead each subunit is approximated as a spherical scatterer with a spherical scattering amplitude *F(q)*, the equation can be written as Eq. 5.

$$I(q) \approx \sum_a \times \sum_b \times F_a(q) F_b(q) \frac{\sin q r_{ab}}{q r_{ab}} \qquad (5)$$

$$= F^2(q) \sum_a \times \sum_b \times \frac{\sin q r_{ab}}{q r_{ab}}$$

where the indices *a* and *b* now run over the subunits. This expression shows that it is enough to know the conformation of the chain and the intensity form factor *F2(q)* of the subunits. The former is obtained from Monte Carlo simulations while the latter can be readily calculated from PDB models using programs such as CRYSOL[7].

Model definition and behavior

The ideal random chain model, which gives the best matching slope in Fig. S4, is not physically reasonable, as an actual chain cannot adopt any random conformation. A real chain cannot overlap with itself and cannot bend at any angle between subunits. In addition, a real chain might attract itself so that non-adjacent subunits tend to stick together. Thus, we constructed a model, which considers these elements, and their parameters are summarized in Table S2 (Fig. S5).

Table S2: Model parameters

N	The number of beads (subunits) per chain.
d	The separation between connecting beads. This is a hard-sphere potential with a radius *d*/2, thus preventing two non-adjacent beads to come closer together than this value.
Θ_{Max}	The maximum angle between beads (*i* - 1), (*i*), and (*i* + 1).
β	Stickiness, defined as $\beta = \varepsilon / RT$, where ε is the bond energy for the sticky interaction and *RT* the thermal energy. A Metropolis condition based on $\exp(-\beta\Delta n)$ is used for each step of the Monte Carlo simulation where Δn bonds are broken. A bond is considered to be formed when the centers of two non-adjacent beads are closer than 1.2*d*. The result is a square-well potential.
$F^2(q)$	The intensity form factor of the subunits.

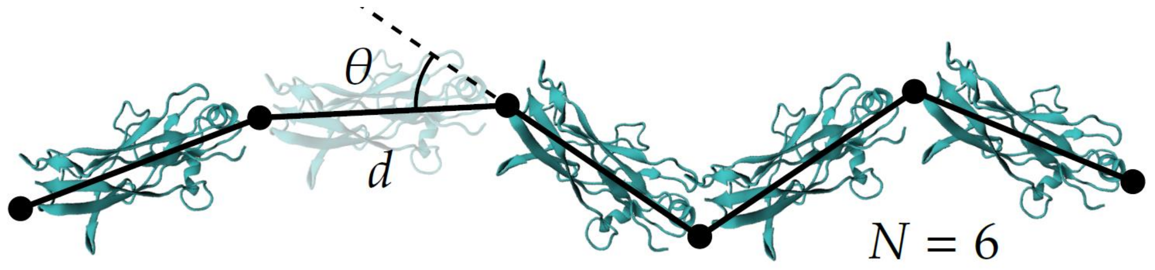

Figure S5 - Illustration of the model, with the NMR subunit structure (from Fig. S1) used as the building block. The molecular representations are made using Visual Molecular Dynamics (VMD) version 1.9.3.[9]

Using Monte Carlo simulations, we can simulate three different fimbriae packing models, supplementing the ideal random coil model (Fig. S6A). In the ideal random coil model, chain overlap with itself was allowed (though it is not physically possible). By introducing self-avoidance in the model, the chain swells because of the repulsive interactions between its different segments. If stickiness is introduced (β >0), the chains shrink again because of the

added attraction. These effects are also observed from the size of the clouds of blue dots, which represent a large number of independent conformations. An example of an entirely random aggregate of independent spheres is also included for comparison.

Using the assumption that one NMR subunit structure is placed along each link of the chain the four different models will result in different X-ray scattering curves (Fig. S6B). As stickiness is increased, there is a smooth transition from the self-avoiding chain behavior to that of random aggregates of unconnected particles. This means that as stickiness becomes very strong, SAXS cannot distinguish chains of subunits from random aggregates of independent subunits. As expected, the Guinier region moves towards higher q (Rg decreases) as stickiness is increased. At the same time the plateau at intermediate q becomes clearer. Both of these changes are analogous to the difference between the A and A+B fimbriae curves (Fig. S4).

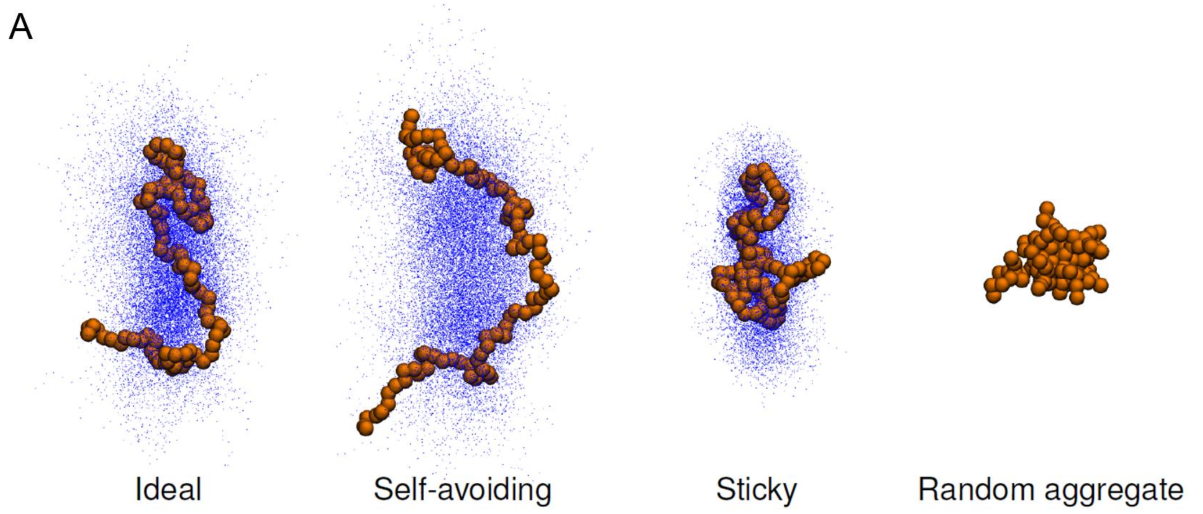

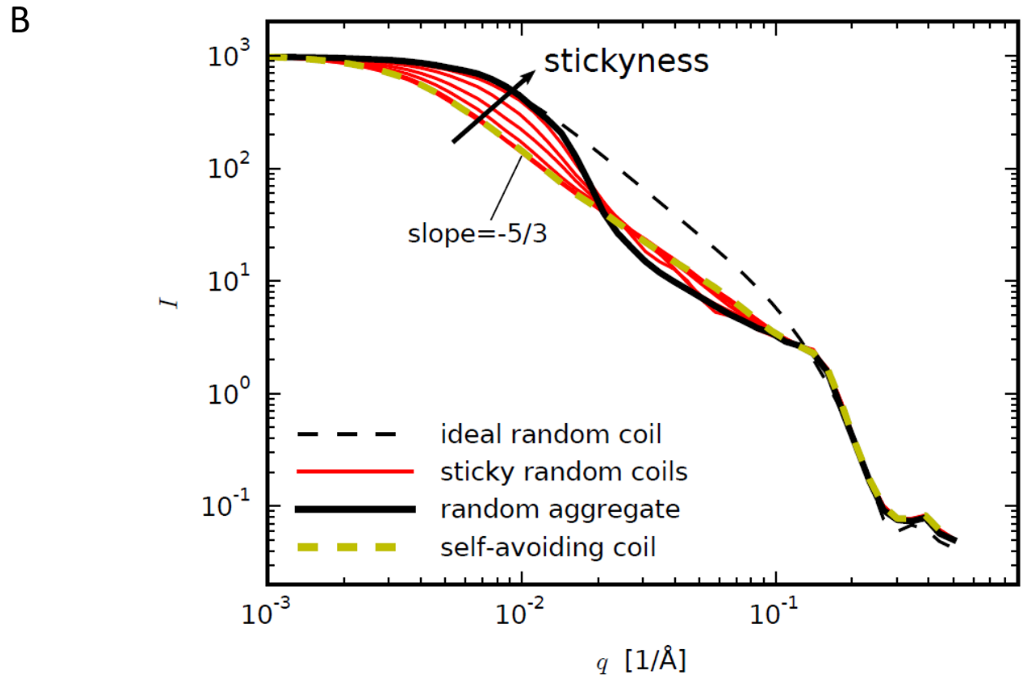

Figure S6.

A Aligned trajectories of simulated ideal random chains, self-avoiding random chains, and sticky self-avoiding chains (β = 1:5). Also shown is a cluster resulting from random (diffusion-limited) aggregation. All clusters contain N = 100 beads with d = 50Å spacing, with $\Theta_{Max} = \pi/2$. F^2 corresponds to the NMR subunit structure. The orange spheres show an arbitrary snapshot, the blue points represent 250 independent frames. Molecular representations are made using VMD version 1.9.3.[9]

B The resulting scattering patterns for varying degrees of stickiness.

In a similar manner as modeling stickiness, we can also model the filament stiffness (Θ_{Max}). within the chain structure and predict its effect on the scattering. As the chain is made stiffer, its scattering pattern transitions from that of the flexible and self-avoiding coil to that of an ideal rod, with a slope of -1 in the log-log plot, as expected (Fig. S7).

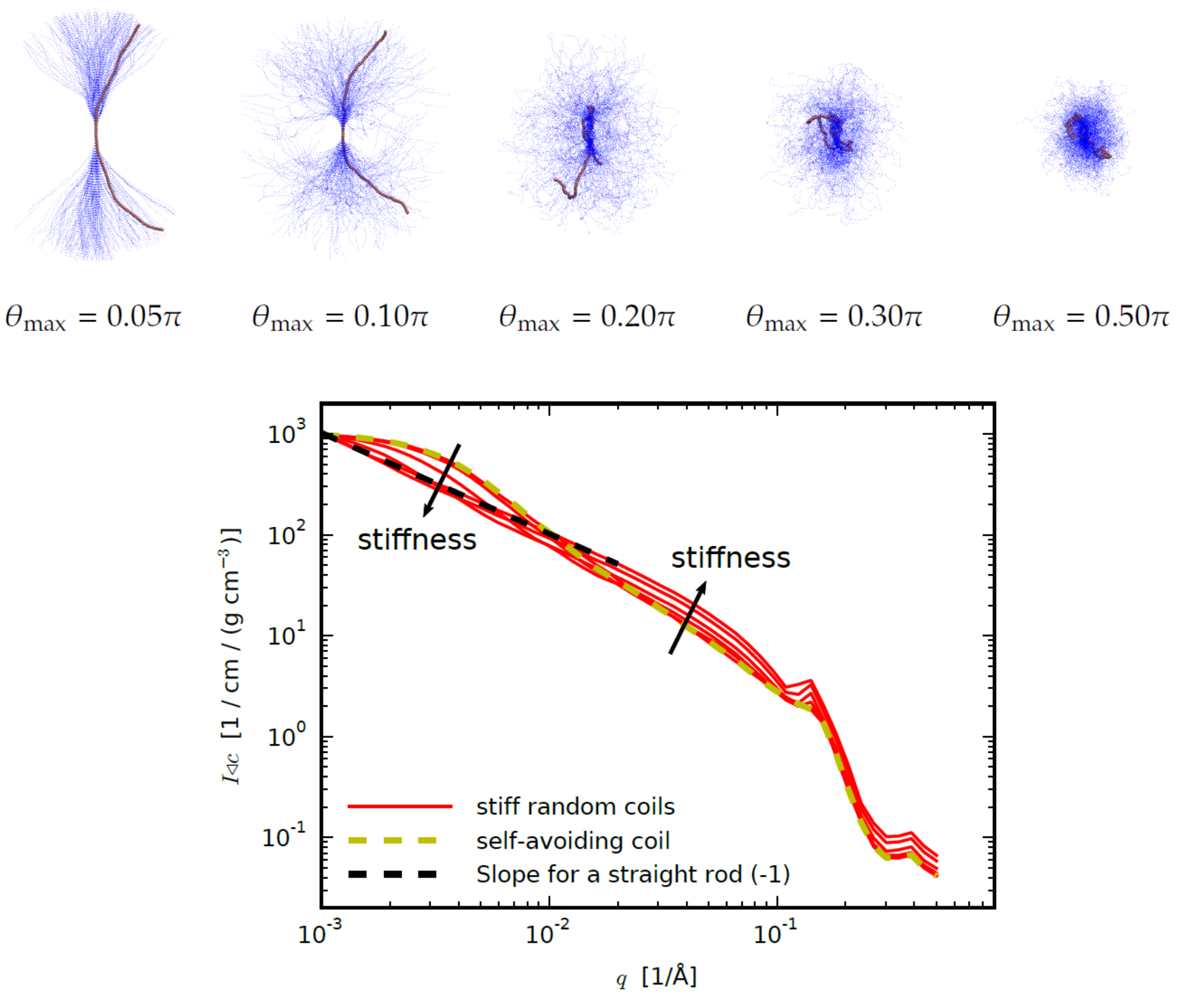

Figure S7: Aligned trajectories of simulated chains of varying stiffness, *N* = 100, *R* = 50 Å, and *β* = 0. Resulting X-ray scattering patterns for the different Θ_{Max} models. Molecular representations are made using VMD version 1.9.3.[9]

References

1. Anderson, K. L. *et al.* An Atomic Resolution Model for Assembly, Architecture, and Function of the Dr Adhesins. *Mol Cell* **15**, 647–657 (2004).

2. Roy, S. P. *et al.* Crystal structure of enterotoxigenic *Escherichia coli* colonization factor CS6 reveals a novel type of functional assembly. *Mol Microbiol* **86**, 1100–1115 (2012).

3. Berry, A. A. *et al.* Structural Insight into Host Recognition by Aggregative Adherence Fimbriae of Enteroaggregative Escherichia coli. *PLoS Pathog* **10**, e1004404 (2014).

4. Jønsson, R. *et al.* Structural and functional studies of Escherichia coli aggregative adherence fimbriae (AAF/V) reveal a deficiency in extracellular matrix binding. *Biochimica et Biophysica Acta (BBA) - Proteins and Proteomics* **1865**, 304–311 (2017).

5. Jędrzejczak, R. *et al.* Structure of DraD invasin from uropathogenic *Escherichia coli* : a dimer with swapped β-tails. *Acta Crystallogr D Biol Crystallogr* **62**, 157–164 (2006).

6. B. E. Warren. *X-ray diffraction*. (Dover Publications, Inc., 1990).

7. Svergun, D., Barberato, C. & Koch, M. H. J. *CRYSOL* – a Program to Evaluate X-ray Solution Scattering of Biological Macromolecules from Atomic Coordinates. *J Appl Crystallogr* **28**, 768–773 (1995).

8. Svergun, D.I. Restoring low resolution structure of biological macromolecules from solution scattering using simulated annealing. *Biophysical journal* **76**, 2879–2886 (1999).

9. Humphrey, W., Dalke, A. and Schulten, K., VMD - Visual Molecular Dynamics. *J. Molec. Graphics* **14,** 33-38 (1996).
